# Post-traumatic stress disorders cases after mild craniocerebral injuries in amateur male athletes

**DOI:** 10.1192/j.eurpsy.2026.11869

**Published:** 2026-07-31

**Authors:** N. Syrmos

**Affiliations:** Aristotle University, Thessaloniki, Greece

## Abstract

**Introduction:**

Introduction-Post-traumatic stress disorder remains a mental pathological situation in which the cause is readily identifiable.

**Objectives:**

Aim-Aim of this study is to present cases with post-traumatic stress disorders cases in amateur male athletes with mild craniocerebral injuries.

**Methods:**

Material-Methods-10 male amateur athletes are presented. Range of age between 18-38 years old .Kind of sports activity:3 cycling activity(30%), 4 combat sports (40%), 3 team sports(30%). All of theme (10-100%) were evaluated, with clinical and radiological exam(ct,mri) plus with the Diagnostic and Statistical Manual of Mental Disorders (DSM-5) diagnostic criteria, prevalence, and presentation of patients with PTSD.

**Results:**

Results-Good results in 9 cases (90%) and moderate in 1 (10%). Collaboration between various medical disciplines is essential in order to achive optimal results and clarify the theory,the etiology, and treatment effectiveness.

**Conclusions:**

Conclusions-Recommendations regarding effective psychotherapy, neurological,psychiatric and pharmacotherapy, emerging treatment, and accurate management are necessary in order to achieve optimal overall treatment.

**Disclosure of Interest:**

None Declared

